# Nutcracker Syndrome Associated With Wilkie’s Syndrome: A Case Report

**DOI:** 10.7759/cureus.85632

**Published:** 2025-06-09

**Authors:** Inês F Lopes, Dulce B Brandão

**Affiliations:** 1 Family Health Unit Sao Miguel, Unidade Local de Saúde Gaia e Espinho, Vila Nova de Gaia, PRT

**Keywords:** abdominal pain, case report, dyspepsia, nutcracker syndrome, superior mesenteric artery syndrome, wilkie’s syndrome

## Abstract

Nutcracker syndrome and Wilkie’s syndrome are rare vascular compression disorders that share a common anatomical etiology, a reduced aortomesenteric angle leading to the compression of the left renal vein and the third portion of the duodenum, respectively. The coexistence of both syndromes is extremely rare. We report the case of a 32-year-old female patient with recurrent left flank pain and gastrointestinal symptoms. The initial imaging revealed a compression of the left renal vein consistent with the nutcracker syndrome. Conservative treatment was initiated. Two years later, she developed postprandial epigastric pain and dyspepsia. Further imaging demonstrated a significant narrowing of the aortomesenteric angle, duodenal compression, and gastric dilatation, confirming the coexistence of Wilkie’s syndrome. Management remained conservative, with multidisciplinary follow-up. This case emphasizes the importance of considering overlapping vascular compression syndromes in patients with evolving abdominal symptoms. Early recognition is crucial to avoid unnecessary investigations and to optimize clinical outcomes.

## Introduction

The abdominal aorta and the superior mesenteric artery (SMA) normally form an angle ranging from 38° to 65° [1]. Wilkie’s syndrome and the nutcracker syndrome (NCS) are rare vascular conditions that result from a reduced angle between the SMA and the abdominal aorta. As the duodenum and the left renal vein lie within the mesenteric angle, either structure may become compressed [2,3].

NCS results from the compression of the left renal vein as it passes between the abdominal aorta and the SMA, leading to an obstruction of the blood flow, and consequently, vascular hypertension [3,4]. The most common clinical manifestations are hematuria, abdominal pain (particularly in the left flank), proteinuria, varicocele, and pelvic congestion syndrome. The latter may include dysmenorrhea, dyspareunia, dysuria, and pelvic pain. Eventually, chronic kidney disease and renal vein thrombosis may occur [1,5,6].

Wilkie’s syndrome, also known as superior mesenteric artery syndrome (SMAS), results from external compression of the third portion of the duodenum between the SMA and the abdominal aorta. This narrowing impairs the passage of the intestinal contents, which may lead to upper gastrointestinal obstruction, which can be chronic, intermittent or acute [3-5]. The symptoms of SMAS include acute or chronic episodic epigastric pain, early satiety, bloating, abdominal distention, esophageal reflux, and postprandial nausea. When the obstruction becomes severe, it may escalate to vomiting, anorexia, and weight loss [2,3,7]. This syndrome is usually associated with rapid growth in children and with rapid weight loss in adults [8].

Even though NCS and SMAS share the same anatomical cause, the coexistence of both conditions is extremely rare [3,5]. We present a case report involving a female patient demonstrating the simultaneous manifestation of NCS and SMAS.

## Case presentation

We present the case of a 32-year-old female patient who presented to her family doctor with episodic abdominal pain, located in the left flank, and radiating to the left pelvic and lumbar regions, with months of evolution. The pain worsened with physical exercise. The patient had daily bowel movements and denied urinary symptoms, dysmenorrhea, or dyspareunia. On physical examination, she was afebrile, normotensive, and had pain on deep palpation of the left iliac fossa and flank, without abdominal tenderness. Her body mass index (BMI) was 19.8 kg/m^2^.

The patient had a history of constipation, anal fissure, and voluntary termination of pregnancy in 2018. She was on combined oral contraceptives and laxative as needed, and had no history of surgical procedures.

A colonoscopy, abdominal ultrasound, blood tests, and urinalysis were done. Blood tests showed no significant findings and renal function was preserved (creatinine level of 0.9 mg/dL). No hematuria or proteinuria were detected on urinalysis. A serrated polyp was excised from the transverse colon, and the abdominal ultrasound revealed no abnormalities. Due to persistent symptoms affecting the patient's daily life, an abdominopelvic CT scan was performed, revealing a narrowing of the left renal vein at the level of its crossing with the SMA, a finding compatible with the nutcracker phenomenon. Additionally, varicose ectasia of the left gonadal vessels was observed. The patient was referred to Vascular Surgery and Nephrology consultations. To rule out other causes of pain, a gynecological ultrasound was also requested, which revealed a uterine fibroid measuring 17 mm. A left hip X-ray did not show any abnormalities. Given the patient's intermittent episodes of abdominal pain, the multidisciplinary team opted for conservative management, with a focus on promoting weight gain to help restore the mesenteric fat pad.

**Figure 1:**
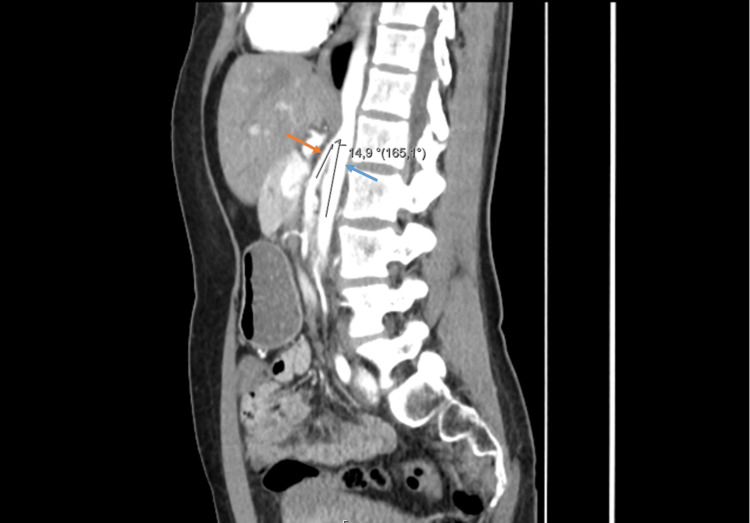
Sagittal CT image at the level of the origin of the SMA (orange arrow) from the aorta (blue arrow) showing a aortomesenteric angle of 14.9° CT: Computed Tomography; SMA: Superior Mesenteric Artery

**Figure 2:**
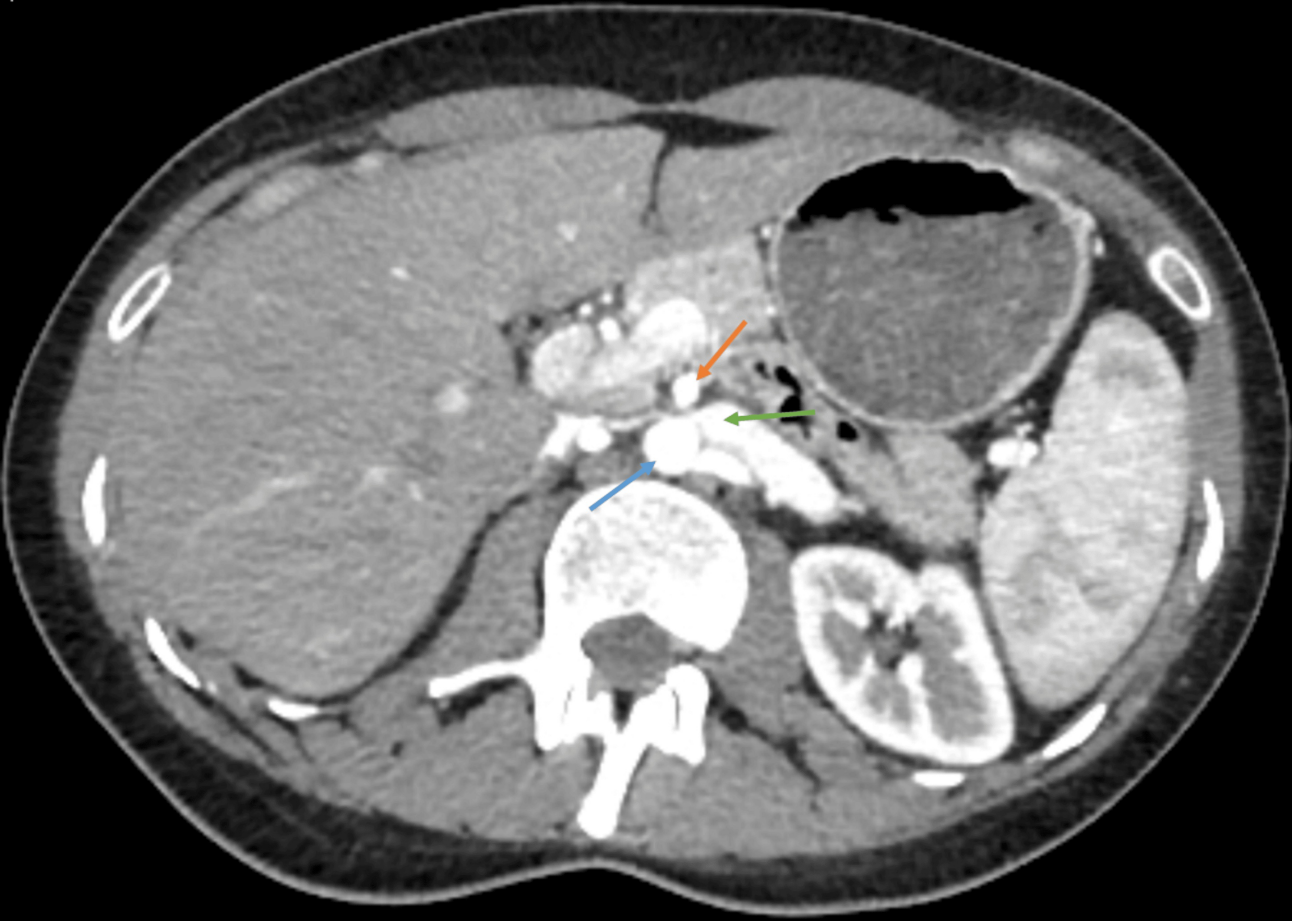
Axial CT image showing narrowing of the left renal vein (green arrow) as it crosses to the left between the SMA (orange arrow) and the aorta (blue arrow) CT: Computed Tomography; SMA: Superior Mesenteric Artery

**Figure 3:**
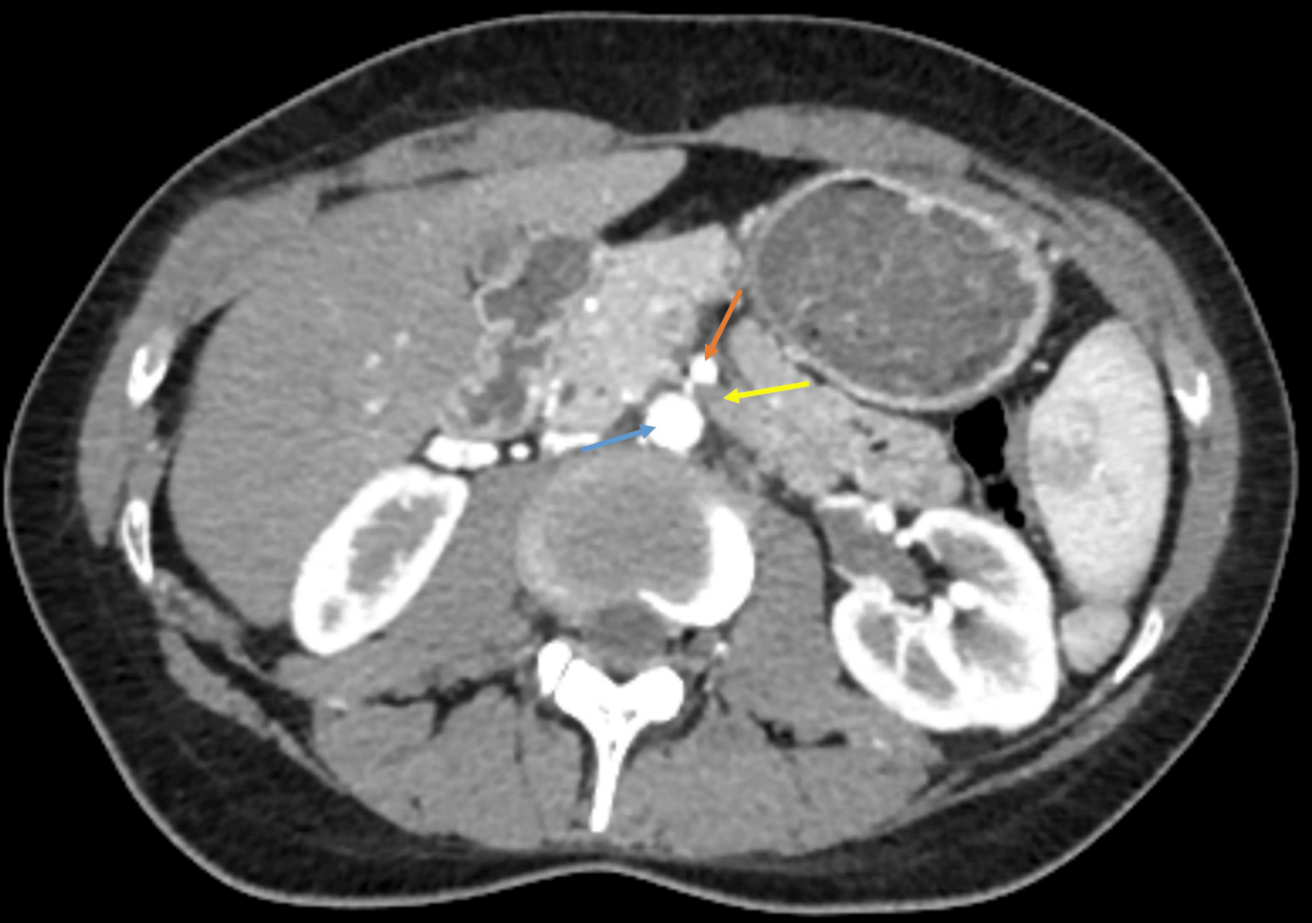
Axial CT image showing duodenal compression (yellow arrow) by the SMA (orange arrow) and the aorta (blue arrow) CT: Computed Tomography; SMA: Superior Mesenteric Artery

**Figure 4:**
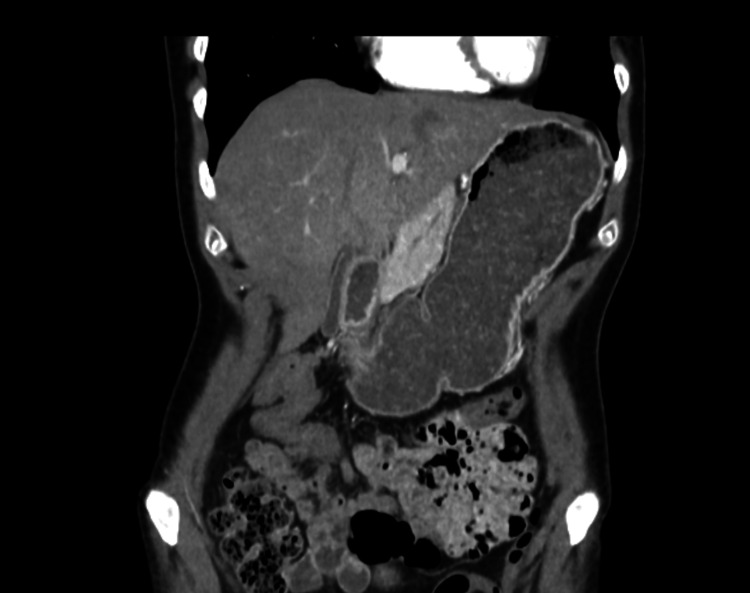
Coronal CT image showing a marked dilated stomach CT: Computed Tomography

Two years later, the patient consulted her family doctor due to recurrent postprandial abdominal pain and dyspepsia. She was prescribed a proton pump inhibitor without any therapeutic response. An upper gastrointestinal endoscopy was requested, revealing a bulging of the gastric mucosa in the body, suggestive of extrinsic compression, and histology showed moderate chronic gastritis. A follow-up abdominal CT scan was conducted, which showed no changes in the stomach but demonstrated a reduced aortomesenteric angle of 14.9° (significantly below the normal range of 38°-65°) (Figure 1), with significant compression of the left renal vein (Figure 2), and the third portion of the duodenum (Figure 3), along with gastric dilatation (Figure 4).

These findings were consistent with both NCS and SMAS. The patient also started follow-up with the gastroenterology team and is currently undergoing conservative management, as there has been no significant weight loss, recurrent vomiting, or episodes of potentially life-threatening obstruction. Since the diagnosis of NCS, the patient has gained six kilograms. Although episodes of abdominal pain and gastrointestinal symptoms continue to occur, they have become less frequent.

## Discussion

The nutcracker phenomenon is the anatomical or radiological finding of left renal vein compression, whereas NCS is defined as the presence of this phenomenon associated with clinical symptoms. NCS is classified into two types: anterior and posterior. The anterior type results from the compression of the left renal vein between the SMA and the abdominal aorta, while the posterior type is associated with a retroaortic left renal vein, an anatomical variant [1]. Most cases are identified in the second or third decade of life, with a higher prevalence in women [1]. The exact prevalence of NCS remains unknown, primarily due to the lack of definitive diagnostic criteria and the wide variability in the clinical presentation [9]. Micro or macroscopic hematuria, often intermittent, is the most frequent sign of NCS, while flank pain is considered the most significant symptom, as it is the main distinguishing feature from other causes of pelvic venous disorders [1,10,11].

The expert panel in the Delphi consensus agreed that NCS is a diagnosis of exclusion and that CT, duplex ultrasound, intravascular ultrasound or magnetic resonance imaging can be used in its diagnosis, since there is currently no universally accepted gold standard diagnostic test for this condition [10]. Depending on the severity of symptoms, treatment may range from conservative management to interventional procedures [1,9]. Conservative management focuses on weight gain to increase retroperitoneal adipose tissue. This approach has been shown to resolve symptoms of NCS in approximately 30% of patients [12]. Interventional approaches range from minimally invasive procedures, such as left renal vein stenting, to more complex surgical techniques, including left renal vein transposition and renal autotransplantation [9]. The expert panel in the Delphi consensus discouraged the use of endovascular stenting as the primary treatment option due to the high risk of complications, such as stent migration [10].

SMAS can present in either an acute or a chronic form. The acute form is characterized by bilious vomiting, abdominal pain, and gastric dilation, which can be potentially life-threatening. The chronic form typically presents with postprandial epigastric pain, along with early satiety, anorexia, and weight loss. Chronic manifestations may result in inadequate food intake, leading to severe weight loss and exacerbation of the syndrome [13]. Similar to NCS, SMAS typically manifests before the age of 40, with a higher prevalence in females and among individuals with a slender or underweight body type [14]. The standard diagnostic exam for SMAS is contrast-enhanced CT imaging. An aortomesenteric angle of less than 25° and an aortomesenteric distance of less than 8 mm are highly suggestive signs of SMAS [13]. In most cases, first-line management is conservative. In the acute setting, this may include intravenous fluids to correct fluid and electrolyte imbalances, the insertion of a nasogastric tube for gastric and duodenal decompression, and positioning the patient in the prone or left lateral decubitus position to relieve duodenal compression and alleviate abdominal pain. Additionally, enteral feeding via a jejunal tube or parenteral nutrition may be used to support weight gain [15]. In the chronic form, conservative management may involve a high-calorie diet designed to restore perivascular adipose tissue and reestablish a normal aortomesenteric angle [3]. Surgical intervention is reserved for cases in which conservative measures fail to produce clinical improvement. Elective surgical management often entails resecting the first duodenal loop and the retrovascular portion of the duodenum, followed by the construction of an anastomosis between the duodenum and the second duodenal loop, both of which are brought anterior to the vascular structures [3]. The optimal length of conservative therapy before surgical intervention remains undefined. Therefore the decision to proceed with surgery should be individualized, taking into account the patient's clinical status, radiological progression, and the judgment of the treating surgeon [15].

Chronic, recurrent abdominal pain without evidence of inflammation or organ dysfunction can pose a diagnostic and therapeutic challenge. This case illustrates a rare cause of recurrent abdominal pain, NCS. The patient initially presented with recurrent episodes of left flank pain in the absence of the most common symptom of the syndrome, hematuria. Two years later, she developed postprandial epigastric pain and dyspepsia, which subsequently led to the diagnosis of concomitant SMAS. Gastrointestinal symptoms are uncommon in NCS, therefore their presence in a patient with NCS should raise suspicion for a possible dual vascular compression and prompt further diagnostic evaluation [14].

## Conclusions

The simultaneous occurrence of NCS and SMAS is rare, even though both conditions share a common anatomical etiology involving compression at the aortomesenteric angle. This case highlights these rare conditions as potential causes of common issues such as abdominal pain and dyspepsia. Therefore, these syndromes should be considered once other, more common causes have been excluded. Increased awareness and understanding of these overlapping syndromes may aid in earlier diagnosis and improve patient outcomes. Early recognition, thorough imaging evaluation, and a multidisciplinary approach are essential for accurate diagnosis and personalized management. Conservative treatment remains the first-line approach for both conditions, with surgical intervention reserved for refractory cases.
